# Educational case: Coccidioidomycosis

**DOI:** 10.1016/j.acpath.2022.100042

**Published:** 2022-08-06

**Authors:** Rachel Brineman, Larry Nichols

**Affiliations:** Mercer University School of Medicine, Macon, GA, USA

**Keywords:** Pathology competencies, Organ system pathology, Respiratory system, Fungal pneumonia, Coccidioidomycosis, Fungal infection in immunosuppression, Special testing, Clinical microbiology

The following fictional case is intended as a learning tool within the Pathology Competencies for Medical Education (PCME), a set of national standards for teaching pathology. These are divided into three basic competencies: Disease Mechanisms and Processes, Organ System Pathology, and Diagnostic Medicine and Therapeutic Pathology. For additional information, and a full list of learning objectives for all three competencies, see https://www.journals.elsevier.com/academic-pathology/news/pathology-competencies-for-medical-education-pcme.^1^

## Primary objective

Objective RS2.10: Fungal pneumonia. Compare and contrast the causative agents, geographic locations, clinical presentation, and pathologic findings in chronic pneumonia caused by fungal organisms.

Competency 2: Organ system pathology; Topic: Respiratory system (RS); Learning goal 2: Pulmonary infection.

## Secondary objectives

Objective FECT2.10: Fungal infection in immunosuppression. Compare and contrast the types of fungal infections that occur in immunosuppressed and immunocompetent patients with respect to the organisms involved, the mechanisms of organ damage, and the resultant clinical manifestations.

Competency 1: Disease mechanisms and processes; Topic: Infectious mechanisms (FECT); Learning goal 2: Pathogenic mechanism of infection.

Objective M5.3: Special testing for fungi and pneumocystis. Explain the basis for the galactomannan and β-glucan tests and how they are utilized to detect fungi and Pneumocystis.

Competency 3: Diagnostic medicine and therapeutic pathology; Topic: Microbiology (M); Learning goal 5: Mycology.

## Patient presentation

A 55-year-old woman presents to the emergency department in February in the northeastern United States with a two-week history of fatigue. This was associated with intermittent low-grade fever, chills, and a mild non-productive cough. She has been on chemotherapy with 5-fluorouracil and leucovorin for metastatic breast cancer, and it has been two weeks since her last one-week round of chemotherapy. Her past medical history is significant for invasive ductal carcinoma of the left breast (positive for estrogen receptors, negative for progesterone receptors and HER2) for which she underwent a mastectomy 15 years ago, followed by tamoxifen therapy. Eleven years ago, she developed metastases of the thoracic spine and ribs, treated with radiation, tamoxifen, and chemotherapy. Six years ago, she developed abdominal lymph node metastases in the porta hepatis associated with biliary obstruction, and again was treated with chemotherapy. Four years ago, she developed hemolytic uremic syndrome attributed to chemotherapy that included mitomycin. She developed end stage renal disease, managed with dialysis for the past four years. She also developed hypothyroidism, treated with levothyroxine. Iliac bone metastases were detected three months ago and treated with chemotherapy (5-fluorouracil and leucovorin).

## Diagnostic findings, Part 1

On examination, the patient appears lethargic, with a temperature of 36.2 °C, pulse 90/min, blood pressure 128/62 mm Hg, respirations 24/min, and oxygen saturation 92% on room air. Her skin is tanned, with a “bronze” hue. She has inspiratory rhonchi and a grade II/VI systolic ejection murmur. Her abdomen is soft and non-tender. Bowel sounds are present. She has no upper or lower extremity edema.

## Questions/discussion points, Part 1

### What is the differential diagnosis based on the history and physical examination?

The differential diagnosis would include metastatic tumor, adverse effect of chemotherapy, or an infection secondary to myelosuppression by chemotherapy. Fatigue is the patient's primary symptom, chief concern, and appropriate focus for the differential diagnosis. Fatigue is a common symptom in patients with metastatic cancer or with anemia secondary to cytotoxic chemotherapy. In this case, the symptom of fatigue by itself could be due to the cytokine response to tumor or to anemia, either from bone marrow replacement by tumor or bone marrow suppression by chemotherapy. Fatigue is also a common symptom of uremia. In this case, fatigue could be partly due to inadequate dialysis or inadequate thyroid hormone replacement. Fatigue could be the way the patient expresses the experience of depression, which would be understandable, given her long battle with metastatic cancer complicated by renal failure requiring dialysis. These potential causes of fatigue are not mutually exclusive.

The patient also has fever, chills, and a cough. Fever can be a manifestation of metastatic cancer, especially with liver metastases. This response is attributed to cytokines such as tumor necrosis factor, although infection is more likely. The patient's fever is intermittent, low-grade and a secondary symptom. The chills are not the rigors associated with high-grade fever and are a secondary symptom. Further questioning might reveal that they represent chilliness from going outdoors in the wintertime. The patient's cough is mild, non-productive and a secondary symptom. Nonproductive cough can be a symptom of interstitial lung disease (such as sarcoidosis or lymphangitic carcinomatosis from breast cancer) or infection. Nonproductive cough due to interstitial lung disease is typically associated with dyspnea, which is lacking in this case. The differential diagnosis for a lung infection with symptoms for two weeks is very different than for a lung infection with symptoms for a day or two; it includes atypical community-acquired bacterial pneumonia (due to *Mycoplasma*, *Chlamydophila* or *Legionella*), viral pneumonia (due to COVID-19, rhinovirus, or metapneumovirus), mycobacterial infection (due to tuberculosis or *Mycobacterium avium*), and fungal infection (due to histoplasmosis or blastomycosis).

The physical examination findings in this case do not fit the classic illness script of any single specific disease. Individual findings suggest various possibilities, some of which broaden, rather than narrow the differential diagnosis. The patient's lethargy raises the specter of brain disease (metastases, infection or depression), but lethargy can also be part of a marked systemic inflammatory response to tumor or infection, or it can be part of uremia. The patient is afebrile, so her fever must have been intermittent. Lack of fever certainly suggests that lethargy is less likely from a marked systemic inflammatory response to infection. Elderly patients often have altered mental status, with confusion progressing to lethargy, without fever, in their response to infection, but this patient is not elderly. The patient's heart rate is in the upper normal range, and her diastolic blood pressure is in the low normal range, which suggests the possibility of mild hypovolemia. However, these hemodynamic parameters are not enough to support a conclusion that the patient's lethargy is from shock.

The patient's respiratory rate is elevated, which could be from pneumonia or compensation for metabolic acidosis. Her oxygen saturation is low, at a threshold cutoff point that some clinicians would use to determine whether a patient with pneumonia should be hospitalized, along with other parameters. Her low oxygen saturation does support the conclusion that she has some lung disease (tumor, infection, edema, fibrosis, emboli, or some combination of these). The majority of patients with pulmonary embolism have dyspnea, which she lacks, and the patient has no extremity edema or erythema to suggest deep venous thrombosis, so embolism is unlikely. Embolism of tumor cells can cause hypoxemia, but this is not common, and unlikely without dyspnea; it would likely require biopsy for diagnosis. The patient has inspiratory rhonchi, which are suggestive of secretions in the bronchi, and this finding supports the conclusion that she has bronchitis. If these secretions could be mobilized, they could be helpful in diagnosing the patient's lung condition.

The hue of the patient's skin suggests several possibilities. The use of the adjective “bronze” for the patient's skin color suggests the possibility of chronic adrenal insufficiency, which is associated with hyperpigmented skin and was once called “bronze Addison's disease.” Adrenal insufficiency could account, at least in part, for the patient's fatigue and low diastolic blood pressure. Adrenal metastases can lead to adrenal insufficiency. The use of the adjective “bronze” for the patient's skin color also raises the possibility of hereditary hemochromatosis, although it is unlikely this disease would have escaped diagnosis given the patient's extensive medical history. Chronic renal disease can produce sallow hyperpigmentation of the skin. The patient's systolic ejection murmur could be a flow murmur associated with anemia, or it could represent thrombus on the aortic valve from marantic endocarditis, which is associated with metastatic cancer. However, the patient lacks evidence of embolic phenomena to corroborate that possibility. Given the patient's 15-year history of metastatic breast cancer, together with the inconclusive abnormalities on physical examination, particularly the lack of fever, pulmonary metastatic disease is a reasonable candidate for the top differential diagnosis based on history and physical examination alone.

## Diagnostic findings, Part 2

The patient is admitted, and her initial laboratory test results are shown in Table 1. Chest radiograph shows an infiltrate in the superior segment of the left lower lobe. Although the patient described her cough as non-productive, sputum is obtained and sent for culture. Then, empirical broad-spectrum antibiotic therapy is initiated with piperacillin-tazobactam, vancomycin and azithromycin. The patient is transfused two units of red blood cells. Over the course of the first three days of hospitalization, the patient has intermittent low-grade fevers, and her cough worsens. She develops a right lower lobe infiltrate and small bilateral pleural effusions. Sputum culture grows normal flora.

**Table 1:** Admission blood test results.

	Patient	Reference Range
White blood cell count	5400/mm^3^	4000–10,000/mm^3^
Segmented neutrophils	75%	40–60%
Bands	6%	0–5%
Lymphocytes	6%	20–40%
Monocytes	7%	2–8%
Eosinophils	4%	1–4%
Basophils	1%	0–1%
Atypical lymphocytes	1%	0–1%
Platelet count	179,000/mm^3^	140,000–440,000/mm^3^
Hemoglobin	7.1 g/dL	11.7–15.7 g/dL
Hematocrit	20.2%	36–46%
Mean corpuscular volume	104.1 fL	80–100 fL
Red cell distribution width	16.25%	11.5–15.4%
Na^+^	138 mEq/L	136-145 mEq/L
K^+^	3.4 mEq/L	3.5–5.1 mEq/L
Cl^−^	101 mEq/L	95–110 mEq/L
HCO_3_^−^	24 mEq/L	21–31 mEq/L
Glucose	125 mg/dL	70–99 mg/dL
Blood urea nitrogen	37 mg/dL	9–20 mg/dL
Creatinine	2.9 mg/dL	0.8–1.5 mg/dL

## Questions/discussion points, Part 2

### How do the admission laboratory test results contribute to making a specific diagnosis?

The white blood cell count is in the normal range. This, along with an absolute neutrophil count of 4374 mm^3^, indicates that this patient is not neutropenic. Neutropenia makes a patient more susceptible to bacterial infection with staphylococci, streptococci, enterococci, *Escherichia coli*, *Pseudomonas aeruginosa* or *Klebsiella pneumoniae*. Neutropenia also makes a patient more susceptible to fungal infection, particularly aspergillosis or mucormycosis. The low hemoglobin level provides an explanation, at least in part, for the patient's primary symptom of fatigue, and prompts a blood transfusion. The only abnormal electrolyte, the slightly low potassium, may indicate a need to slightly adjust the dialysate for her renal replacement therapy. The mildly elevated glucose may represent a nonspecific stress response from cortisol secreted by the adrenal cortex, or it could simply be post-prandial. The anemia revealed by the admission laboratory testing provides an etiology for the patient's fatigue, which is her chief concern. The normal absolute neutrophil count makes infection due to neutropenia unlikely.

### Why would the lung disease progress on broad-spectrum antibiotic therapy?

Progression of pneumonia despite broad-spectrum antibiotic therapy can be due to antibiotic resistance, which can develop during the course of appropriate therapy covering the pathogen, or it can be due to a pathogen not covered by the antibiotic therapy. Pneumonia from COVID-19, for instance, can progress despite broad-spectrum antibacterial therapy. Fungal pneumonia is generally not covered by the broad-spectrum antibiotic therapy for bacterial pneumonia. Progression of pneumonia despite broad-spectrum antibiotic therapy can also be due to non-infectious pneumonitis, from an autoimmune process or a drug reaction, or from tumor such as lymphangitic carcinomatosis from metastatic melanoma or breast cancer.

## Diagnostic findings, Part 3

**Fig. 1:**
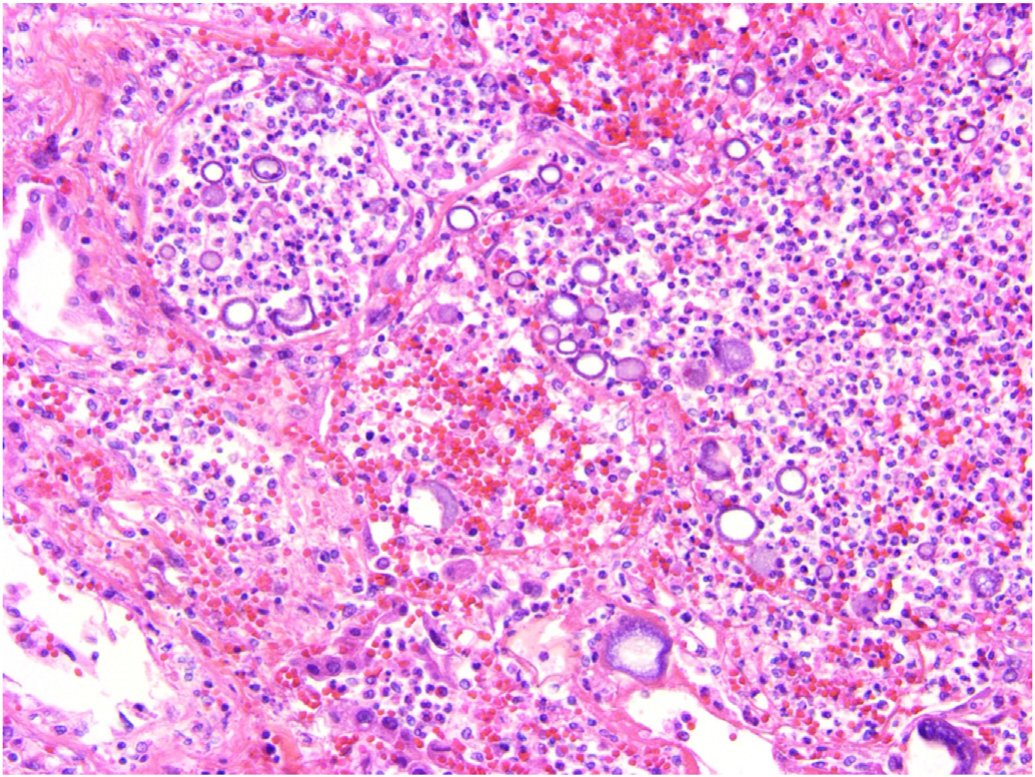
Alveoli filled with neutrophils, red blood cells and variably sized, round, mostly empty, basophilic-walled spherules (H&E, X10).

**Fig. 2:**
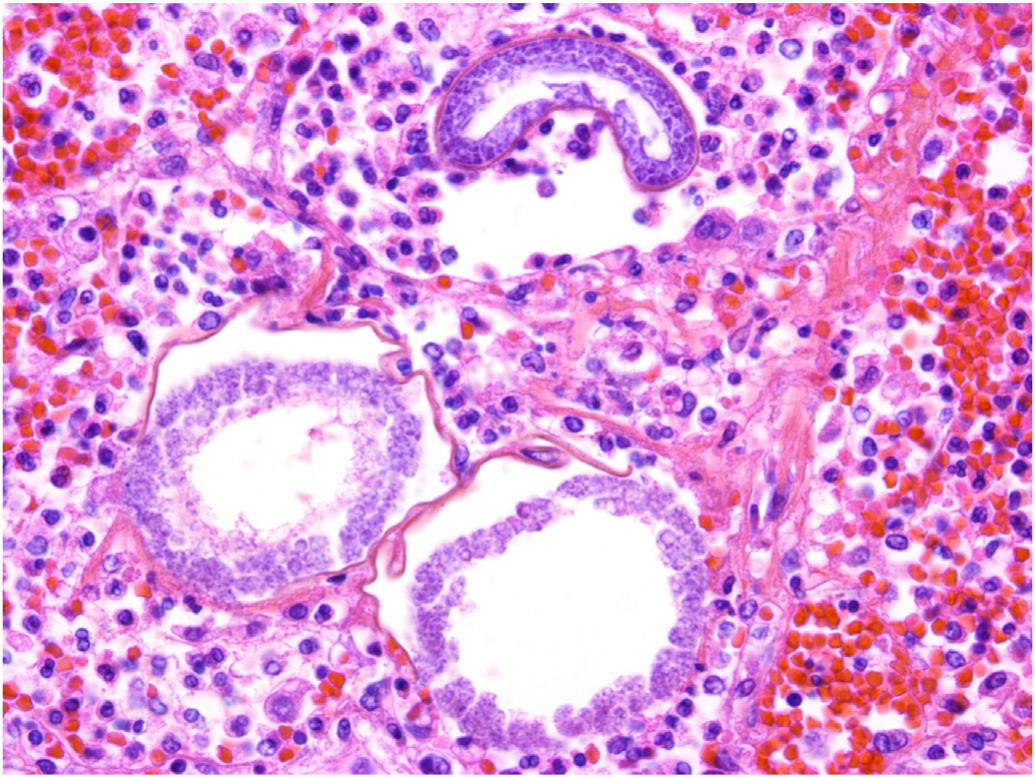
Three ruptured spherules containing lightly basophilic endospores, associated with hemorrhage and an inflammatory infiltrate of neutrophils, lymphocytes and macrophages (H&E, X40).

On rounds in the hospital, the question arises whether the bilateral lung infiltrates might be metastatic tumor. The medical team discusses how aggressive the patient and her family want to be in pursuit of a lung disease diagnosis. The medical student on the team has spent more time speaking with the patient than others on the team. The student points out that the patient's tan is from a two-week trip to Tucson, Arizona, and that she is not at all ready to “just give up.” A decision is reached to do a video-assisted thoracoscopic lung biopsy in pursuit of a definitive diagnosis. The lung biopsy is shown in Fig. 1 and Fig. 2.

## Questions/discussion points, Part 3

### Describe the lung biopsy findings shown in Figs. 1 and 2

Fig. 1 shows alveoli filled with neutrophils, red blood cells and variably sized, round, mostly empty, basophilic-walled spherules. Fig. 2 shows three ruptured spherules containing lightly basophilic endospores, associated with hemorrhage and an inflammatory infiltrate of neutrophils, lymphocytes and macrophages.

## Diagnostic findings, Part 4

Coccidioidomycosis is diagnosed by the pathologist on the basis of the lung biopsy findings.

## Questions/discussion points, part 4

### What fungi cause pneumonia in immunocompetent patients?

In North America, there are three major endemic mycoses: histoplasmosis, blastomycosis and coccidioidomycosis. All three can present as community-acquired pneumonia in immunocompetent patients.^2^ Each one of these mycoses is present in only a specific geographic region, as shown in Table 2.

**Table 2:** Geographically limited endemic mycosis that cause pneumonia in immunocompetent patients.

Mycosis	Organism	Region	Morphology
Histoplasmosis	*H. capsulatum*	Eastern half of the US and of Mexico	Small intracellular budding yeast forms
Blastomycosis	*B. dermatitidis, B. gilchristii*	Eastern half of the US and of Canada	Large yeasts with broad-based budding
Coccidioidomycosis	*C. immitis, C. posadasii*	Southwest US and most of Mexico	Spherules and spores

Patients sometimes present with one of these types of pneumonia outside of the endemic area. In such cases, the travel history is crucial to making the diagnosis, as illustrated in this educational case. Medical students should also note the crucial role they can play. They often have more time than other members of the health care team and should use it to listen carefully and empathetically to the patient, eliciting more of the often-neglected social history, dietary history, and travel history.

### What fungi cause pneumonia in immunocompromised patients?

Immunocompromised patients can present with fungal pneumonia due to the three fungi mentioned that cause pneumonia in immunocompetent patients (*Histoplasma*, *Blastomyces* and *Coccidioides*). In addition, there are four opportunistic fungal pathogens that cause pneumonia in immunocompromised patients: *Aspergillus* species, *Cryptococcus*, *Mucor* species and *Pneumocystis*.^3^ These do not all share the same risk factors, as shown in Table 3.

**Table 3:** Opportunistic mycosis that cause pneumonia in immunocompromised patients.

Mycosis	Organism	Major Risk Factor	Morphology
Aspergillosis	*A. fumigatus, A. niger*	Neutropenia	Regular septate hyphae with acute angle branching
Cryptococcosis	*C. neoformans, C. gattii*	Deficient cell-mediated immunity	Variably sized budding yeasts with large capsules
Pneumocystis pneumonia	*P. jirovecii*	Deficient cell-mediated immunity	Collapsed cysts and small “trophozoites”
Mucormycosis	*Rhizopus* sp.*, Mucor* sp.	Diabetes mellitus	Large empty twisted hyphae with rare any-angle branching

### How does the morphology of *Coccidioides* differ from other fungi that cause pneumonia?

Histoplasmosis features small intracellular budding yeast forms, as described in Table 2, but these are often few in number, difficult to see on routine hematoxylin and eosin (H&E) stain in immunocompetent patients, and may be recognized only on methenamine silver stain. In profoundly immunocompromised patients, such as those with advanced acquired immunodeficiency syndrome (AIDS), *Histoplasma* are more numerous and easier to appreciate on H&E stain. Blastomycosis has large yeast forms with broad-based budding, often in small numbers. *Aspergillus* infecting lung has regular septate hyphae with branching at acute angles, as described in Table 3, sometimes tightly packed in radial array. Cryptococcal species have variably sized, budding yeast forms with large capsules that appear as surrounding cleared spaces on routine H&E stain. The fungi of pneumocystis pneumonia are rarely visible on H&E stain, but appear as collapsed cysts, which may resemble helmets or teacups, on methenamine silver stain, or small “trophozoites” on Giemsa stain. The fungal species that cause mucormycosis (*Mucor*, *Rhizopus*, *Rhizomucor*, *Apophysomyces*, *Cunninghamella*, *Lichtheimia*, and *Saksenaea*) have large, twisted, often empty-appearing, pauciseptate hyphae, with rare branching at random angles, sometimes right angles.

### What are the clinical manifestations of fungal pneumonia?

Fungal pneumonias, in general, are more like other pneumonias than they are different. The most common signs and symptoms of fungal pneumonia are cough and fever, the same as bacterial pneumonia.^3^^,^^4^ In a study comparing symptoms of fungal pneumonia with other respiratory illnesses, cough was the most common symptom of coccidioidomycosis (in 31.4% of patients), but also the most common symptom of pneumococcal pneumonia (in 33.2%).^4^ Patients may also have pleuritic chest pain, dyspnea and fatigue as manifestations of fungal pneumonia, the same as bacterial pneumonia. Although the symptoms of fungal pneumonia such as fever, cough and dyspnea are very similar to bacterial or viral pneumonia, they tend to be less severe, with a slower onset, and are more chronic, on average, in patients whose pneumonia is fungal. In the study comparing symptoms of fungal pneumonia with other respiratory illnesses, fever was less common in fungal pneumonia (2.6–9.4%) than in patients with influenza (18.5%) or pneumonia (12.6–16.3%).^4^ Most patients are in an overlapping middle range, but the more the symptoms are mild and chronic, with insidious onset, the more likely a pneumonia is fungal. In the study comparing fungal and other pneumonias, enlarged lymph nodes were more common in fungal pneumonia (4–9%) than in other types of pneumonia (0.4–2%), likely reflecting the more chronic nature of fungal pneumonia.^4^

Blood testing may show less leukocytosis with fungal pneumonia than with bacterial pneumonia, but this difference is not much help in determining that a patient has fungal pneumonia. Coccidioidomycosis causes peripheral blood eosinophilia in 25–30% of cases, but parasitic infections (such as ascariasis), and Churg-Strauss syndrome (eosinophilic granulomatosis with polyangiitis) are more common causes of lung disease with eosinophilia. Chest radiograph patterns can be helpful.^5^ Fungal pneumonias less often have a lobar pattern than bacterial pneumonia. *Pneumocystis* and *Cryptococcus* often produce an interstitial pattern of pneumonia.^5^ Histoplasmosis and coccidioidomycosis may produce a diffuse nodular pattern of pneumonia.^5^

Specific fungal pneumonias are more like each other than they are different. The most common symptoms of coccidioidomycosis in the study comparing fungal and other respiratory illnesses were cough (31.4%), dyspnea (17.5%), malaise/fatigue (15.3%), chest pain (14.5%), arthralgia (12.9%) and fever (9.43%).^4^ The most common symptoms of blastomycosis were cough (13.1%), chest pain (10.1%), dyspnea (9.7%) and arthralgia (9.7%).^4^ The most common symptoms of histoplasmosis were dyspnea (14.6%), cough (14%), chest pain (11.2%) and arthralgia (10.3%).^4^

The most common radiographic manifestation of acute coccidioidomycosis (in 75% of cases) is consolidation (segmental or lobar), usually unilateral and often at the lung base.^6^ The second most common radiographic appearance is small-intermediate size, 0.5–3 cm nodules, typically bilateral, and up to 40% of patients have lymphadenopathy.^6^ The most common radiographic manifestation of acute blastomycosis (in 56% of cases) is also consolidation (segmental or lobar), unilateral or bilateral, more often in the upper lobes.^6^ The second most common finding in acute blastomycosis (in 31%) is lung masses, up to 10 cm.^6^ Symptomatic acute histoplasmosis is most often radiologically manifested as consolidation, segmental or lobar, unilateral or bilateral, but it may appear as nodular opacities, and it is associated with more lymphadenopathy than blastomycosis or coccidioidomycosis.^6^ Most cases of all three major mycoses endemic in North America are asymptomatic or manifest as undiagnosed, self-limited disease.

Fungal pneumonias that are more common in immunocompromised patients often have more severe and more varied clinical manifestations than the three major mycoses endemic in North America. Aspergillosis has a particularly wide spectrum of disease, ranging from rapidly fatal invasive aspergillosis in neutropenic patients to aspergillomas colonizing lung cavities in immunocompetent patients.^7^ Patients with invasive aspergillosis typically have prolonged profound neutropenia, cough, hemoptysis, dyspnea and pleuritic chest pain. Patients with an aspergilloma typically have only a chronic cough. Although cryptococcosis is thought of as a disease of the immunocompromised, more than half of patients with pulmonary cryptococcosis are immunocompetent.^8^ The symptoms of pulmonary cryptococcosis are fever, cough, dyspnea and chest pain. Pneumocystis pneumonia typically presents with the subacute onset of dyspnea, a non-productive cough and low-grade fever.^9^ Pulmonary mucormycosis occurs most often in patients with neutropenia or uncontrolled diabetes, who typically present with some combination of fever, cough, hemoptysis, dyspnea or chest pain.^10^

The radiographic appearances of invasive pulmonary aspergillosis include nodules, consolidative lesions, and wedge-shaped infarcts.^11^ One characteristic radiological finding of invasive pulmonary aspergillosis on computed tomography is the halo sign, a central nodule surrounded by ground-glass opacity that represents hemorrhage. Another characteristic finding is the air crescent sign, a crescent-shaped radiolucency within a nodular opacity representing retracted infarcted lung.^11^ Pulmonary cryptococcosis characteristically produces nodules, which may be single or multiple and are often peripheral.^8^ *Pneumocystis* characteristically produces bilateral perihilar interstitial infiltrates with increasing involvement of lung fields and homogeneity over time. Computed tomography may reveal ground-glass opacification.^9^ Like cryptococcosis and aspergillosis, pulmonary mucormycosis most often produces nodular lesions, but one characteristic finding on computed tomography is a reversed halo sign, which is a rounded area of ground-glass opacity surrounded by a ring of consolidation.^12^

### What is the pathogenesis of coccidioidomycosis?

*Coccidioides* species fungi are adapted to living in alkaline soil in an arid hot desert climate.^13^^,^^14^ They grow in soil as mycelia, which septate and produce alternating atrophic segments and spores called arthroconidia. The septations are fragile, and with minimal soil disturbance from wind or digging, arthroconidia become airborne. Infection is almost always from inhalation of arthroconidia.^13^ In the lungs of infected patients, arthroconidia transform into spherules, which then internally divide into endospores. Large mature spherules rupture, releasing endospores, which become spherules, resulting in exponential growth of the pathogen.^13^^,^^14^ Both the innate and adaptive immune systems are involved in the immunological response, which is sequential, multifaceted, very complex and only partly elucidated.^3, 13.^ People of African or Filipino descent are more susceptible to severe coccidioidomycosis.^14^^,^^15^ It has been suggested that genetic influences linked to ABO blood groups and HLA alleles likely play a role in this susceptibility, although these may be surrogate markers for socioeconomic determinants of health.^15^ The endospores and spherules in an infected patient are not contagious, so coccidioidomycosis is not spread from person to person.

### Besides lung biopsy, how can pulmonary coccidioidomycosis be diagnosed?

The detection of *Coccidioides* in a clinical specimen by microscopy or culture is the diagnostic standard, but not often quick and easy.^16^ *Coccidioides* grow on routine clinical microbiology media in two to seven days, but they grow as mycelia, producing infectious arthroconidia, which may infect unsuspecting microbiology laboratory workers.^17^ In fact, *Coccidioides* are designated agents of bioterrorism, so suspected and established cultures of *Coccidioides* should be handled using Biosafety Level 3 containment.^17^ This makes it especially important to know when to suspect coccidioidomycosis, which is especially difficult outside the endemic area. The diagnosis of coccidioidomycosis is usually made by blood testing for antibodies, although serology can be false negative early in the immune response or in patients who are immunocompromised.^16^ Immunocompetent patients usually develop antibodies within one to three weeks after disease onset. Most clinical laboratories do enzyme immunoassays to detect these antibodies and the results are generally available within 24 hours.^18^ Immunodiffusion is more specific but less sensitive than enzyme immunoassay. Immunodiffusion is typically performed to confirm the diagnosis and allay concerns about false-positive results.^16^

Beta-D-glucan and galactomannan are polysaccharide fungal cell-wall derivatives that can be detected in serum, bronchoalveolar lavage and cerebrospinal fluid to diagnose invasive fungal disease.^19^^,^^20^ Beta-D-glucan is a component of the cell wall of many fungal pathogens, including *Candida*, *Aspergillus*, *Pneumocystis*, and *Histoplasma*. It is being increasingly used for early diagnosis of invasive fungal diseases, especially candidiasis, but the primary value has been in diagnosing fungal invasion, not identifying which fungus is invading. For coccidioidomycosis, beta-D-glucan assay demonstrates a sensitivity of 43.9%, specificity of 91.1%, positive predictive value of 81.8%, and a negative predictive value of 64.1%, which is comparable to the assays in diagnosing other invasive mycoses.^21^ Galactomannan antigen test has been most used to diagnose invasive aspergillosis.^20^ However, an antigen enzyme immunoassay specific for *Coccidioides* that detects galactomannan antigens in blood, urine, and other clinical specimens is also available.^20^^,^^22^ This galactomannan has cross reactivity with other endemic fungi, so it lacks the high specificity needed for an ideal method of diagnosis.^22^ Recently, an approach combining antibody detection by immunodiffusion and antigen detection has demonstrated a diagnostic accuracy of 95.4% for the diagnosis of progressive coccidioidomycosis, compared to 93.6% for immunodiffusion antibody test alone, and 89.1% for pathology or culture.^23^ Research is ongoing to develop better tests for coccidioidomycosis.^16^^,^^18^^,^^23^

### What is the treatment and prognosis for coccidioidomycosis?

Most cases of pneumonia due to *Coccidioides* should not be treated with antifungal agents.^16^ Antifungal agents have toxicity and are not recommended for treatment unless the patient is at risk of complications or already shows signs of complicated or disseminated infection. Fluconazole and itraconazole are the most commonly recommended antifungal drugs, with an exception of during pregnancy. Pregnant women needing antifungal therapy are normally treated with intravenous amphotericin B, although fluconazole can be considered during the second and third trimesters.^16^ Amphotericin B is also recommended for severe or rapidly progressing coccidioidomycosis in transplant patients, until the patient has stabilized, when fluconazole can be substituted and continued indefinitely.^24^ If antifungals are given, serial complement fixation titers should be done for at least two years because antifungal treatment has been associated with delayed dissemination. Antifungal therapy is often discontinued after three to twelve months if symptoms resolve, chest radiography shows stabilization, and complement fixation titers stabilize.^16^Antifungal therapy with fluconazole is recommended for patients with human immunodeficiency virus infection, continued as long as their CD4 lymphocyte count is less than 250 cells/mm^3^.^24^ Most patients with coccidioidomycosis recover without sequelae and have a good prognosis.

## Teaching points

• Histoplasmosis, blastomycosis and coccidioidomycosis are endemic, geographically limited fungal infections that often present as community-acquired pneumonia.
• Because of the geographic limitation of these mycoses, when a patient with one of them presents in a non-endemic region, the patient's travel history can be the key to a life-saving diagnosis.
• *Aspergillus*, *Cryptococcus*, *Pneumocystis*, and *Mucor* species are opportunistic fungal pathogens that can present as severe pneumonia in immunocompromised patients.
• Coccidioidomycosis is endemic in the desert Southwest United States and most of Mexico.
• African Americans, Filipino Americans, pregnant women, and immunocompromised patients are at risk for severe coccidioidomycosis.
• The diagnosis of coccidioidomycosis can be made by a pathologist, who identifies spherules in tissue from biopsy or autopsy.
• The diagnosis of coccidioidomycosis can be made by culture of *Coccidioides*, but culture is dangerous because the infectious form of the fungus grows in the laboratory.
• Serologic identification of antibodies by enzyme immunoassay confirmed by immunodiffusion is the method most often used to make a diagnosis of coccidioidomycosis.
• Assays of beta-D-glucan or galactomannan (GM) can be used to diagnose invasive fungal disease but have limited utility for the diagnosis of coccidioidomycosis.
• Coccidioidomycosis most often requires no antifungal treatment.
• When treatment is needed, this is most often with oral fluconazole or itraconazole.
• In pregnant women, coccidioidomycosis is treated with intravenous amphotericin.
• Coccidioidomycosis usually has a good prognosis.

## Funding

The article processing fee for this article was funded by an Open Access Award given by the Society of ‘67, which supports the mission of the Association of Pathology Chairs to produce the next generation of outstanding investigators and educational scholars in the field of pathology. This award helps to promote the publication of high-quality original scholarship in *Academic Pathology* by authors at an early stage of academic development.

## Declaration of competing interest

None declared.

## References

[1] Knollmann-Ritschel B.E.C., Regula D.P., Borowitz M.J., Conran R., Prystowsky M.B. (2017). Pathology competencies for medical education and educational cases. Acad Pathol.

[2] Ashraf N., Kubat R.C., Poplin V. (2020). Re-Drawing the maps for endemic mycoses. Mycopathologia.

[3] Li Z., Lu G., Meng G. (2019). Pathogenic fungal infection in the lung. Front Immunol.

[4] Benedict K., Kobayashi M., Garg S., Chiller T., Jackson B.R. (2020). Symptoms in blastomycosis, coccidioidomycosis, and histoplasmosis versus other respiratory illnesses in commercially insured adult outpatients, United States, 2016-2017. Clin Infect Dis.

[5] Herring W. (2016).

[6] Kunin J.R., Flors L., Hamid A., Fuss C., Sauer D., Walker C.M. (2021). Thoracic endemic fungi in the United States: importance of patient location. Radiographics.

[7] Moldoveanu B., Gearhart A.M., Jalil B.A., Saad M., Guardiola J.J. (2021). Pulmonary aspergillosis: spectrum of disease. Am J Med Sci.

[8] Setianingrum F., Rautemaa-Richardson R., Denning D.W. (2019). Pulmonary cryptococcosis: a review of pathobiology and clinical aspects. Med Mycol.

[9] Bateman M., Oladele R., Kolls J.K. (2020). Diagnosing Pneumocystis jirovecii pneumonia: a review of current methods and novel approaches. Med Mycol.

[10] He J., Sheng G., Yue H., Zhang F., Zhang H.L. (2021). Isolated pulmonary mucormycosis in an immunocompetent patient: a case report and systematic review of the literature. BMC Pulm Med.

[11] El-Baba F., Gao Y., Soubani A.O. (2020). Pulmonary aspergillosis: what the generalist needs to know. Am J Med.

[12] Jain A., Knoll B., Lim S., Kleinman G., Epelbaum O. (2021). For whom the atoll tolls: targeting the reversed halo sign. Am J Med.

[13] Johnson R.H., Sharma R., Kuran R., Fong I., Heidari A. (2021). Coccidioidomycosis: a review. J Invest Med.

[14] Kollath D.R., Miller K.J., Barker B.M. (2019). The mysterious desert dwellers: Coccidioides immitis and Coccidioides posadasii, causative fungal agents of coccidioidomycosis. Virulence.

[15] Brown J., Benedict K., Park B.J., Thompson G.R. (2013). Coccidioidomycosis: epidemiology. Clin Epidemiol.

[16] Herrick K.R., Trondle M.E., Febles T.T. (2020). Coccidioidomycosis (valley fever) in primary care. Am Fam Physician.

[17] Ampel N.M. (2010). The diagnosis of coccidioidomycosis. F1000. Med Rep.

[18] Ampel N.M. (2020). Coccidioidomycosis: changing concepts and knowledge gaps. J Fungi (Basel).

[19] Zangeneh T.T., Malo J., Luraschi-Monjagatta C. (2015). Positive (1-3) B-d-glucan and cross reactivity of fungal assays in coccidioidomycosis. Med Mycol.

[20] Hites M., Goicoechea Turcott E.W., Taccone F.S. (2016). The role of galactomannan testing to diagnose invasive pulmonary aspergillosis in critically ill patients. Ann Transl Med.

[21] Thompson G.R., Bays D.J., Johnson S.M., Cohen S.H., Pappagianis D., Finkelman M.A. (2012). Serum (1->3)-β-D-glucan measurement in coccidioidomycosis. J Clin Microbiol.

[22] Malo J., Luraschi-Monjagatta C., Wolk D.M., Thompson R., Hage C.A., Knox K.S. (2014). Update on the diagnosis of pulmonary coccidioidomycosis. Ann Am Thorac Soc.

[23] Kassis C., Durkin M., Holbrook E., Myers R., Wheat L. (2021). Advances in diagnosis of progressive pulmonary and disseminated coccidioidomycosis. Clin Infect Dis.

[24] Galgiani J.N., Ampel N.M., Blair J.E. (2016). 2016 Infectious diseases society of America (IDSA) clinical practice guideline for the treatment of coccidioidomycosis. Clin Infect Dis.

